# The Impact of Repeated Rounds of Mass Drug Administration with Diethylcarbamazine Plus Albendazole on Bancroftian Filariasis in Papua New Guinea

**DOI:** 10.1371/journal.pntd.0000344

**Published:** 2008-12-09

**Authors:** Gary J. Weil, Will Kastens, Melinda Susapu, Sandra J. Laney, Steven A. Williams, Christopher L. King, James W. Kazura, Moses J. Bockarie

**Affiliations:** 1 Washington University School of Medicine, St. Louis, Missouri, United States of America; 2 Case Western Reserve University School of Medicine, Cleveland, Ohio, United States of America; 3 Papua New Guinea Institute of Medical Research, Madang, Papua New Guinea; 4 Smith College, Northampton, Massachusetts, United States of America; Centers for Disease Control and Prevention, United States of America

## Abstract

**Background:**

This study employed various monitoring methods to assess the impact of repeated rounds of mass drug administration (MDA) on bancroftian filariasis in Papua New Guinea, which has the largest filariasis problem in the Pacific region.

**Methodology/Principal Findings:**

Residents of rural villages near Madang were studied prior to and one year after each of three rounds of MDA with diethylcarbamazine plus albendazole administered per World Health Organization (WHO) guidelines. The mean MDA compliance rate was 72.9%. Three rounds of MDA decreased microfilaremia rates (Mf, 1 ml night blood by filter) from 18.6% pre-MDA to 1.3% after the third MDA (a 94% decrease). Mf clearance rates in infected persons were 71%, 90.7%, and 98.1% after 1, 2, and 3 rounds of MDA. Rates of filarial antigenemia assessed by card test (a marker for adult worm infection) decreased from 47.5% to 17.1% (a 64% decrease) after 3 rounds of MDA. The filarial antibody rate (IgG_4_ antibodies to Bm14, an indicator of filarial infection status and/or exposure to mosquito-borne infective larvae) decreased from 59.3% to 25.1% (a 54.6% decrease). Mf, antigen, and antibody rates decreased more rapidly in children <11 years of age (by 100%, 84.2%, and 76.8%, respectively) relative to older individuals, perhaps reflecting their lighter infections and shorter durations of exposure/infection prior to MDA. Incidence rates for microfilaremia, filarial antigenemia, and antifilarial antibodies also decreased significantly after MDA. Filarial DNA rates in *Anopheles punctulatus* mosquitoes that had recently taken a blood meal decreased from 15.1% to 1.0% (a 92.3% decrease).

**Conclusions/Significance:**

MDA had dramatic effects on all filariasis parameters in the study area and also reduced incidence rates. Follow-up studies will be needed to determine whether residual infection rates in residents of these villages are sufficient to support sustained transmission by the *An. punctulatus* vector. Lymphatic filariasis elimination should be feasible in Papua New Guinea if MDA can be effectively delivered to endemic populations.

## Author Summary

Lymphatic filariasis (LF) is a deforming and disabling disease that is caused by parasitic worms that are transmitted by mosquitoes. While a number of countries have initiated LF elimination programs based on mass drug administration (MDA), relatively little good information is available on the impact of MDA on filariasis prevalence and incidence rates in populations. This study assessed the impact of three rounds of MDA (with single doses of diethylcarbamazine and albendazole) on filariasis infection rates in villages in Papua New Guinea, which has the largest filariasis problem in the Pacific region. MDA dramatically reduced rates for all filariasis infection markers tested. These included microfilaremia (parasites in blood that are necessary for transmission of the infection), filarial antigenemia (a marker for adult worm infection), anti-filarial antibodies (which indicate infection or heavy exposure to the parasite), and parasites in mosquitoes that transmit the infection. In addition to curing existing infections, MDA also reduced new infection rates in the study population to very low levels. These results suggest that it should be possible to eliminate LF in Papua New Guinea if MDA can be effectively delivered to endemic populations.

## Introduction

Lymphatic filariasis (LF) is a deforming and disabling infectious disease that causes elephantiasis and hydroceles. The infection affects some 120 million people in an estimated 83 countries in tropical and subtropical regions, with an estimated 1.2 billion individuals at risk [1]. Most LF is caused by *Wuchereria bancrofti*, a nematode parasite that is transmitted to humans by mosquitoes. The World Health Assembly passed a resolution in 1997 that called for global elimination of LF as a public health problem (WHA Resolution 50.29, see www.filariasis.org). The World Health Organization (WHO) developed a plan for elimination that is based on selective diagnosis to identify endemic areas followed by repeated, annual cycles of mass drug administration (MDA) of antifilarial medications [1],[2]. The most recent summary from WHO reported that approximately 1.9 billion doses of MDA were distributed to more than 500 million individuals between 2000 and 2007 [3]. Thus, the Global Programme to Eliminate Lymphatic Filariasis (GPELF) is the largest infectious disease intervention program attempted to date based on MDA. Applied field research is needed to validate the GPELF strategy and to test methods for measuring the impact of MDA. Recent papers have reported encouraging data on the impact of MDA with diethylcarbamazine (DEC) plus albendazole on various filariasis parameters in Egypt and emphasized the importance of compliance in MDA programs [4],[5]. However, more information is needed from areas with different epidemiological and ecological parameters, e.g. mosquito vectorial capacity, transmission intensity, baseline infection rates, and climate.

Bancroftian filariasis is a major public health problem in Papua New Guinea [6]. With approximately 39% of its population infected, the country has some of the highest LF infection rates in the world [7], despite the fact the principal vectors there (*Anopheles punctulatus* group, which also transmit malaria) are less efficient for filariasis transmission than *Culex* or *Aedes* mosquitoes that serve as vectors in other endemic regions [6],[8]. At this time, Papua New Guinea has more filariasis cases than any other country in the Pacific region [9]. Although it is the only country in the region that has not yet fully implemented a national LF elimination program, the Papua New Guinea Department of Health has recently initiated a MDA program for elimination in several provinces. In some areas, MDA will overlap with the distribution of insecticide-treated bednets for malaria control. This overlap in public health interventions may benefit LF elimination efforts, as suggested by reports from the Solomon Islands where DDT spraying (used for malaria control) reduced LF transmission [10].

Several studies conducted over the past 10 years have assessed the impact of MDA on LF infection parameters in Papua New Guinea. For example, a study performed in 14 villages in East Sepik Province found that four rounds of MDA with DEC plus ivermectin or with DEC alone (drug regimens that have been superseded by the current WHO recommendation of single dose DEC plus albendazole) dramatically decreased microfilaremia (Mf) prevalence rates and transmission parameters in areas with high and moderate baseline infection and transmission rates [11]. Other studies in offshore island communities in Papua New Guinea found dramatic decreases in Mf and/or filarial antigenemia rates following MDA or community distribution of DEC alone or DEC with albendazole [12],[13]. We now report results from a community study that was designed to evaluate the efficacy of repeated rounds of MDA with DEC plus albendazole (distributed according to WHO guidelines [14]) on a number of filariasis infection parameters. Results of this study should be useful for those responsible for planning and implementing national LF elimination programs in Papua New Guinea and in other areas where filariasis is transmitted by *Anopheles* mosquitoes.

## Methods

### Study site

The study was performed in 4 villages in the Usino-Bundi district, Madang Province, Papua New Guinea. The villages are located approximately 40 km southwest of the provincial capital, Madang town. The study villages were located at least 2 km from untreated filariasis-endemic villages. This is the estimated flight range of the local mosquito vector [15]. Villages were mapped and censuses were conducted prior to the first round of MDA and annually thereafter. Two Infection prevalence surveys were performed before the first round of MDA, and this was repeated approximately 12 months after each of three annual rounds of MDA.

### Field procedures and MDA

Field teams comprised of trained nurses and field technicians met with community leaders and held outdoor community education sessions to inform people about the health significance and biology of LF, the planned MDA program, and the importance of blood tests for monitoring the impact of MDA. Annual follow-up meetings were held to communicate preliminary results and to provide community members with opportunities to ask questions about the project.

Community liaison personnel mobilized village residents to participate in the study. Children <2 years of age, children who weighed <10 kg, pregnant women, and people with severe chronic illness or acute illness with fever were excluded from the study. We reassessed eligibility for treatment each year. For example, women who were pregnant in year 1 were eligible for the study in later years if they were not pregnant. Survey teams enrolled subjects in the late afternoon by obtaining oral informed consent from adults and recording demographic information. Enrollment of children required their assent and consent from at least one parent. The population was surveyed twice prior to the first round of MDA. The first pre-MDA survey (pre-MDA-A, which collected day blood samples for antigen and antibody testing only without microfilaria testing) was performed approximately 2 years prior to the first round of MDA. The second pre-MDA survey (pre-MDA-B, with blood for Mf, antigen, and antibody testing) was performed together with the first round of MDA (with blood collection just prior to treatment).

MDA comprised the WHO-recommended regimen of a single oral dose of diethylcarbamazine citrate (6 mg/kg body weight) with albendazole (400 mg regardless of weight). This was repeated once per year for a total of three years. Project staff directly observed ingestion of antifilarial medications. Some people agreed to receive MDA but refused to provide blood samples. MDA compliance rates were estimated by dividing the number of people who ingested DEC with albendazole by the number of village residents in the census who were at least 2 years of age. This is similar to “observed coverage” in GPELF guidelines [14]. However, persons ineligible for treatment (e.g., pregnant women) are included in the denominator for our compliance calculations but not in the GPELF “observed coverage”.

### Blood tests for filariasis infection or exposure

All tests were performed in the Papua New Guinea Institute of Medical Research Laboratories near Madang. Microfilaremia was assessed by membrane filtration of 1 ml of venous blood collected between 9 p.m. and 12 midnight [16]. The Mf prevalence rate was calculated by dividing the number of people who were Mf positive by the number of people tested for Mf. Few blood samples were collected from children younger than 6 years of age, and results from these samples are not included in this report. We also calculated community microfilaria load (CMFL) as a measure of the total amount of Mf present in study communities as previously described [17]. CMFL is defined as the antilog of the [sum of log (X+1)/N]–1, where X is the MF count in positive subjects and N is the number of people tested for microfilaremia.

Circulating filarial antigenemia (CFA) was assessed with a rapid-format antigen card test (Filariasis Now, Binax Inc., Portland, Maine, USA) [18],[19]. Test instructions call for testing 100 µl of whole blood. We tested 70 µl of plasma, which is the approximate volume of plasma in 100 µl of whole blood. Card tests were read visually at 10 minutes. IgG_4_ antibodies to recombinant filarial antigen Bm14 in human plasma were detected by ELISA as previously described [20]. Sera were tested in duplicate, and borderline samples were retested. Prior studies have shown that this test is sensitive and specific for infection or heavy exposure to filarial parasites [20]–[22].

### Molecular xenomonitoring

Mosquitoes were collected with CDC light traps (without dry ice) placed in houses (1 night per house) in study villages over 4 months period before MDA commenced and for 4 months starting 6 to 9 months following each round of MDA. Two pre-MDA mosquito collections were conducted. The first (pre-MDA-A) was conducted approximately 12 to 18 months prior to the first round of MDA; the second (pre-MDA-B) was 1 to 5 months prior to the first round of MDA.

Blood-engorged, gravid, or semi-gravid *An. punctulatus* group mosquitoes (all of which had recently ingested blood) were identified by morphology and sorted into pools by household. *W. bancrofti* DNA was detected in mosquito pools by PCR as previously described in detail [23]–[25], except mosquitoes were macerated by vortexing with ball bearings [26] instead of by mortar and pestle. Mosquito infection rates (maximum likelihood estimates with 95% confidence intervals) were calculated with PoolScreen 2.02 software [27].

### Data entry and statistical methods

Data entry was performed with Visual FoxPro software with field limits and double data entry. We used the SPSS v.14 software package (SPSS, Chicago, IL) for statistical analysis. The chi-square test was used to assess the significance of differences in proportions.

### Ethical clearance

This study (including the oral consent process) was reviewed and approved by institutional review boards at Washington University School of Medicine and Case Western Reserve University and the Medical Research Advisory Committee of the Papua New Guinea Department of Health. The sponsor of the study (Division of Microbiology and Infectious Diseases, NIAID, NIH, Bethesda, MD) also reviewed the study protocol to ensure compliance with GCP standards. Study personnel informed prospective study participants about the study by reading them a consent document in the local language. Oral consent was documented on case report forms. Participation by children required consent from at least one parent and the child's assent. As noted above, some subjects agreed to take anti-filarial medications but refused blood tests.

## Results

### Pre-MDA human infection parameters

Table 1 shows baseline infection indicators by age group just prior to the first round of MDA (pre-MDA-B survey data). Mf prevalence increased dramatically up to age 20 years, with slightly higher rates in older age groups. The mean±SD of the number of Mf per ml in 106 microfilaremic subjects was 610±1073 (median 161; range 1–5,960). CFA and Bm14-specific IgG_4_ antibody rates increased dramatically from age 6–10 years to 21–30 years, with slight increases (CFA) or little change (Bm14) in older age groups. Antibody and CFA rates were much higher than Mf rates in all age groups. Persons with Mf or filarial antigenemia had a significantly higher antibody rate (81.9%, n = 279) than persons with negative Mf and CFA tests (37.6%, N = 287) (*P*<0.001). Antibody rates were higher than CFA rates in younger age groups (≤30 years of age, *P*<0.001), but CFA rates were similar to antibody rates in older people (difference not significant). Filarial infection rates (Mf and CFA) were significantly higher in males than in females (Table 2). Males also had higher antifilarial antibody rates, but the difference was not statistically significant. Gender differences in infection and antibody rates were much more striking in adults than in children 6 to 15 years of age, although the same trends were present in children.

**Table 1:** Filariasis infection parameters by age in Usino villages just prior to the first round of mass drug administration.

Age Range	Mf (%)	CFA (%)	Bm14 (%)
**6–10**	3/125 (2.4)	22/120 (18.3)	43/120 (35.8)
**11–15**	7/90 (7.8)	25/87 (28.7)	40/87 (46.0)
**16–20**	13/60 (21.7)	27/60 (45)	37/60 (61.7)
**21–30**	31/131 (24.4)	78/128 (60.9)	95/129 (73.6)
**31–40**	24/80 (30.0)	52/79 (65.8)	54/79 (68.4)
**41–50**	24/49 (29.2)	34/47 (72.3)	37/48 (77.1)
**51+**	13/37 (35.1)	27/37 (73.0)	26/37 (70.3)
**Total**	106/571 (18.6)	265/558 (47.5)	332/560 (59.3)

Abbreviations: Mf, microfilaremia; CFA, circulating filarial antigenemia; Bm14, antibodies to recombinant filarial antigen Bm14.

Data shown are the number of positive tests and the total number of tests performed (% positive).

**Table 2:** Filariasis infection parameters by gender in Usino villages just prior to the first round of mass drug administration.

Gender	Mf Prev (%)	CFA Prev (%)	Bm14 (%)
**Females 6–15**	2/89 (2.2)	15/87 (17.2)	33/87 (37.9)
**Males 6–15**	8/126 (6.3)	32/120 (26.7)	50/120 (41.7)
***P*** ** value**	0.28	0.15	0.69
**Females >15**	32/182 (17.6)	99/181 (54.7)	117/181 (64.6)
**Males >15**	64/174 (36.8)	119/170 (70)	132/172 (76.7)
***P*** ** value**	<0.001	0.005	0.02
**All Females >5**	34/271 (12.5)	114/268 (42.5)	150/268 (56.0)
**All Males >5**	72/300 (24.0)	151/290 (52.1)	182/292 (62.3)
***P*** ** value**	0.001	0.024	0.126

Abbreviations: Mf, microfilaremia; CFA, circulating filarial antigenemia; Bm14, antibodies to recombinant filarial antigen Bm14.

Data shown are the number of positive tests and the total number of tests performed (% positive). Numbers in the first column refer to age in years.

### Effects of MDA on filariasis infection parameters in humans

The mean MDA compliance rate over the 3 rounds of MDA was 72.9% (Table 3). However, compliance rates were much lower in children 2–5 years of age compared to those in older individuals.

**Table 3:** Mass drug administration (MDA) compliance rates in Usino, Papua New Guinea.

Age (yr)	Round 1		Round 2		Round 3	
	Census	MDA Compliance %	Census	MDA Compliance %	Census	MDA Compliance %
**2–5**	147	29.9	146	39	130	23.1
**>5**	825	75.3	854	82.8	841	81.7
**≥2**	972	68.4	1000	76.4	971	73.9

MDA had dramatic effects on all filariasis parameters examined (Table 4). Over the three annual rounds of MDA, microfilaremia (Mf prevalence rates and CMFL) decreased more rapidly and to lower levels than CFA or antibody rates. Antigenemia and antibody prevalence rates in young children decreased more after MDA than rates for the total study population.

**Table 4:** Filariasis parameters before and after mass drug administration (MDA) in Usino villages.

		Mf (%)	CMFL	CFA (%)	Bm14 (%)
**All ages**	**Pre-MDA-A**	n/a	n/a	334/627 (53.3)	411/683 (60.2)
	**Pre-MDA-B**	106/757 (18.6)	1.45	265/558 (47.5)	332/560 (59.3)
	**MDA-1**	58/696 (8.3)	0.44	243 692 (35.1)	276/696 (39.7)
	**MDA-2**	24/714 (3.4)	0.16	175/695 (25.2)	337/693 (48.8)
	**MDA-3**	7/529 (1.3)	0.03	93/543 (17.1)	138/550 (25.1)
	**% Decrease**	**93.0**	**97.9**	**64.0**	**54.6**
**Age <11**	**Pre-MDA-A**	n/a	n/a	26/130 (20.0)	52/137 (38.0)
	**Pre-MDA-B**	3/125 (2.4)		22/120 (18.3)	43/120 (35.8)
	**MDA-1**	1/138 (0.7)		14/137 (10.1)	17/138 (12.3)
	**MDA-2**	0/129 (0)		3/124 (2.4)	21/124 (16.3)
	**MDA-3**	0/129 (0)		3/105 (2.9)	9/109 (8.3)
	**% Decrease**	**100**		**84.2**	**76.8**

Data shown are the number of positive tests and the total number of tests performed (% positive).

The pre-MDA-A survey did not include Mf testing. CMFL was calculated for all ages only.

Pre-MDA-B values were used as the baseline for calculating % decrease for rates after MDA.

Abbreviations: Mf, microfilaremia; CMFL, community microfilarial load (see Methods); CFA, circulating filarial antigenemia; Bm14, antibodies to recombinant filarial antigen Bm14.

**Table 5:** Filarial antigenemia and Bm14 antibody rates in subjects with microfilaremia before and after mass drug administration (MDA).

	CFA (%)	Bm14 (%)
**Pre-MDA-B**	98/104 (94.2)	80/104 (76.9)
**MDA-1**	56/58 (96.6)	41/58 (70.7)
**MDA-2**	21/23 (91.3)	18/23 (78.3)
**MDA-3**	6/7 (85.7)	6/7 (85.7)

Data shown are the number of subjects with positive tests and the number of Mf-positive subjects tested (% positive) before the first round of MDA and approximately 12 months after each round of MDA.

Abbreviations: Mf, microfilaremia; CFA, circulating filarial antigenemia; Bm14, IgG_4_ antibodies to recombinant filarial antigen Bm14.

Seven of 529 subjects tested had Mf one year after the third round of MDA. Mf counts in these subjects ranged from 1–52 Mf/ml (mean 18.0±20.5; median 9). These subjects tended to have high Mf counts prior to MDA. Only one of the subjects with Mf detected after MDA round 3 had not received MDA; two had been treated once, one had been treated twice, and two had been treated three times.

### Sensitivity of the CFA and Bm14 antibody tests in Mf-positive subjects before and after MDA

Table 5 shows CFA and antibody rates in Mf-positive subjects detected in study villages before MDA and after each round of MDA. These rates did not change significantly after MDA. *P* values for differences in CFA and Bm14 antibody rates (for samples from Mf-positive subjects) in different years of the study were 0.60 and 0.78, respectively (chi-square).

### Longitudinal observations on microfilaremia after MDA

Longitudinal Mf data were available for 104 subjects who were initially Mf positive and who had repeat Mf testing approximately one year after one or more rounds of MDA until Mf clearance was achieved. Mf clearance rates were 76% (79/107), 95.2% (99/104), and 98.1% (102/104) after 1, 2, or 3 rounds of treatment, respectively; 2 subjects had persistent microfilaremia after 3 rounds of treatment. Subjects with higher baseline Mf counts tended to require more rounds of MDA to completely clear Mf. The geometric mean and median Mf counts for 79 people who cleared Mf after one round of MDA were 64.8 and 89 (range, 1–3813); geometric mean and median Mf counts for 25 subjects who failed to clear Mf after one round of MDA were 604 and 833 (range 11–5279). The difference in baseline Mf counts between the two groups was statistically significant (*P*<0.001 by the Mann Whitney U test).

Microfilaria incidence rates in persons who were Mf-negative just prior to MDA were 1.7% (7/408), 0.4% (2/555), and 0.2% (1/472) after MDA rounds 1, 2, and 3, respectively. Note that Mf incidence data were not available for the period between the two pre-MDA surveys. Despite this, the decrease in Mf incidence rates after MDA was statistically significant (*P* = 0.013 by chi square). The mean Mf count in persons with Mf incidence was 86.2±112 Mf/ml (median 26, range 7–353). Of course, some of these cases may represent “pseudo-incidence” due to technical problems with Mf detection or clerical errors. Most people with Mf incidence had positive antigen (7/10) and antibody (8/10) tests one year prior to the appearance of Mf. Four subjects with microfilaremia at baseline were Mf-negative after MDA but positive again with counts ranging from 47 to 68 Mf/ml one year later. Such cases of temporary Mf clearance following treatment were not counted in clearance or incidence results shown above.

### Decreased antibody and antigenemia incidence rates after MDA

Table 6 shows Bm14 antibody and CFA incidence events and rates for the total study population and for children 6–15 years of age. Incidence rates for the 2 year period between the 2 pre-MDA surveys were high. Pre-MDA incidence rates in children <16 years of age were not significantly different from those in older people. Incidence rates decreased dramatically after MDA. Incidence rates for the 2 year period after the first round of MDA were significantly lower than those for the two year period between the pre-MDA-A and pre-MDA-B surveys (Table 6). Incidence rates for antifilarial antibodies and CFA decreased by 75% and 63% in the total study population (and by 68% and 70% in children), respectively, after 3 rounds of MDA.

**Table 6:** Incidence events and incidence rates for anti-filarial antibodies and filarial antigenemia before and after mass drug administration (MDA).

	Survey	Bm14 Incidence (rate, %)	CFA Incidence (rate, %)
**All ages**			
	**Pre-MDA-B**	21/154 (6.8)	11/120 (4.6)
	**MDA-1**	5/164 (3.0)	2/201 (1.0)
	**MDA-2**	7/213 (3.3)	3/287 (1.0)
	**MDA-3**	3/181 (1.7)	5/291 (1.7)
**Age <16**			
	**Pre-MDA-B**	14/83 (8.4)	7/56 (6.3)
	**MDA-1**	4/98 (4.1)	1/118 (0.8)
	**MDA-2**	4/131 (3.1)	0/143 (0)
	**MDA-3**	3 /111 (2.7)	3/155 (1.9)

Incidence events for each test were defined as positive tests in people who had tested negative in the prior survey and who had never had a positive test in prior years. This analysis is restricted to persons with data available for consecutive years. The pre-MDA-B incidence data are for a 2 year interval (with annual incidence rates shown in parentheses). Other incidence data are for one year intervals. Annual incidence rates for the 2 year period between pre-MDA-A and pre-MDA-B were significantly higher than rates for the two years following the first round of MDA (*P*<0.001 by chi-square for all comparisons).

Abbreviations: CFA, circulating filarial antigenemia; Bm14, IgG_4_ antibodies to recombinant filarial antigen Bm14.

### Effect of MDA on filarial DNA rates in mosquitoes

573 pools of recently fed (blood fed, gravid, or semigravid) mosquitoes (3,729 mosquitoes) were tested for filarial DNA by PCR (Table 7). The mosquitoes were collected from 114.6±16.9 houses per year (approximately 70% of the houses in study villages). The mean number of recently fed mosquitoes per pool was 6.5±6.6 (median 4.0; range 1–28). The high baseline rate of filarial DNA in mosquitoes just prior to MDA (15.1% in the pre-MDA-B survey) decreased rapidly after MDA (Table 7). The 92.3 % decrease in the filarial DNA rate after 3 rounds of MDA was similar to the 93% decrease in the Mf prevalence rate observed in the human population.

**Table 7:** Mosquito collections and filarial DNA rates in mosquitoes before and after mass drug administration (MDA).

Survey	No. Pools	Mosq. per Pool (SD)	No. Mosquitoes Tested	Filarial DNA Rate (95% CI)
**Pre-MDA-A**	97	6.8 (6.1)	565	11.4 (10.87–21.25
**Pre-MDA-B**	119	7.5 (7.3)	892	15.1 (11.7–19.2)
**MDA-1**	118	6.2 (6.4)	726	3.7 (2.2–5.6)
**MDA-2**	130	6.7 (6.8)	926	4.8 (3.3–6.7)
**MDA-3**	100	6.2 (6.4)	620	1.02 (0.4–2.2)
**Totals**	573	6.5 (6.6)	3,729	

Filarial DNA was detected in mosquitoes by PCR, and rates (maximum likelihood with 95% CI) were estimated by PoolScreen. The filarial DNA rate in mosquitoes collected in the MDA-3 survey (6 to 9 months after the third round of MDA) represented a 92.3% decrease from the rate in mosquitoes collected during the pre-MDA-B survey (just prior to the first round of MDA).

## Discussion

This is the first detailed report on the effects of repeated rounds of MDA with DEC and albendazole on filariasis infection parameters in an area with *Anopheles* transmission. The study site had baseline filarial infection rates that were high in the global context but moderate for Papua New Guinea. Baseline infection rates were higher in older age groups and higher in males than in females. These trends probably reflect increased cumulative exposure in older people and differences in exposure between males and females. Since the gender differences were much more striking in adults, these could also be related to biological factors such as hormone levels.

The performance of the ICT antigen test and the Bm14 antibody test require some comment before we address the impact of MDA on filariasis parameters in this study. The ICT test detected filarial antigenemia in a high percentage of untreated Mf-positive subjects (detected by membrane filtration of 1 ml of venous blood), and this sensitivity was maintained after MDA. This is in contrast to a recent study from Kenya that reported decreased sensitivity of the ICT test in Mf carriers (detected by the counting chamber method with 0.1 ml of finger prick blood) after 2 rounds of MDA [28]. The Bm14 antibody test was less sensitive than the CFA test in Mf-positive subjects in the present study, and it was also somewhat less sensitive than previously reported [20],[22]. However, both the CFA test and the Bm14 antibody test were much more sensitive than Mf detection for detecting filariasis activity in the study communities, and this is consistent with prior reports [5],[29]. Antibody rates in children <11 years of age were much higher than Mf or CFA rates, both before and after MDA. This supports the strategy of testing sentinel populations of young children for antifilarial antibodies as a means of assessing recent filariasis activity in communities [30].

Population MDA compliance rates were very good throughout the 3-year study period. However, this required a lot of effort, with multiple visits to the study villages and labor-intensive recruitment of village residents. It might be easier to achieve high compliance rates in a national MDA program that was not linked to collection of venous blood. MDA compliance rates were low in children <6 years of age. This may have reflected parents' concerns about blood tests in their young children. We believe that the national LF elimination program in Papua New Guinea will need to develop new strategies to achieve high MDA compliance in young children. Information campaigns should emphasize the dual benefits of MDA on LF and soil-transmitted helminth infections.

MDA dramatically reduced all filariasis infection parameters in the study villages. As in earlier studies, Mf rates in people and parasite DNA rates in mosquitoes fell more rapidly than CFA or antibody rates [5],[31],^32^. While low residual filarial DNA rates in mosquitoes indicate the presence of Mf carriers in communities following MDA, this does not necessarily mean that significant LF transmission will continue in these areas. PCR can detect DNA from dead filarial parasites in mosquitoes [33], and most Mf taken up by anopheline vectors do not survive to become infective larvae (L3) [34],[35].

CFA and anti-filarial antibody rates fell more rapidly after MDA in children <11 years of age than in the total study population; this may be because infection intensities and years of infection/exposure tend to be lower in young children than in older individuals. Although infection rates decreased in children after MDA, many young children had positive CFA and/or anti-filarial antibody tests after 3 rounds of MDA. Of course, these children had been exposed to the parasite for years prior to MDA. Children born after LF transmission has been interrupted should not have positive CFA or antibody tests [30]. Surveillance activities to verify interruption of transmission should focus on testing young children. Mosquito monitoring provides a non-invasive means of detecting residual infections in communities if the number of young children available for testing is small.

This study provided interesting longitudinal data on effects of MDA on Mf clearance in individuals and on incidence rates for different filariasis parameters. Mf clearance rates in this study after one or more annual doses of DEC with albendazole were higher than those reported from clinical trials performed in Sri Lanka and Egypt [36]–[38]. However, while all subjects in the clinical trials had high baseline Mf counts, all Mf carriers were considered in current community-based study. The current study also found that Mf clearance rates after MDA were lower in persons with high baseline Mf counts. The incidence data are very exciting, because they demonstrated that MDA significantly decreased the incidence of Mf, CFA, and antifilarial antibodies in the study population. This is the first study that has documented decreased filariasis incidence rates following MDA. However, incidence events observed after MDA-3 suggest that 3 rounds of MDA was not sufficient to completely eliminate LF transmission in this setting.

The impact of three rounds of MDA in the current study was at least as impressive as that recently reported from Egypt (Giza governorate) with the same MDA regimen, although this “high prevalence” study area in Egypt had lower LF infection rates before MDA (11.5% Mf, 19.0% CFA, and 3.07% mosquito DNA) and higher MDA compliance rates than our study site in Papua New Guinea [5]. This suggests that the encouraging results reported from Egypt can be replicated in areas with very different epidemiological parameters. Changes in infection parameters following MDA must be considered in the context of the local mosquito vector. *An. punctulatus* is a less efficient LF vector than *Cx. pipiens* (the principal LF vector species in Egypt). We do not know the minimum requirements for sustained transmission of *W. bancrofti* by *An. punctulatus*. However, Tisch et al recently reported that LF parameters continued to decrease in villages in a different area of Papua New Guinea (in East Sepik Province, approximately 300 km from the Usino study site) for at least 5 years after Mf rates had been reduced to low levels by 5 rounds of MDA with DEC and ivermectin [32]. It is possible that three rounds of DEC with albendazole (which reduced the Mf rate to 1.3% with a 97.9% decline in CMFL) would have been sufficient to reduce LF transmission rates to unsustainable levels in the Usino study area. The study villages received a fourth round of MDA in 2006; long term follow-up studies will be needed to determine whether four rounds of MDA have interrupted LF transmission in this area. For the time being, the results from Usino are quite encouraging. Taken together with earlier studies, they suggest that LF elimination should be feasible in Papua New Guinea and other endemic areas with *Anopheles* transmission if MDA can be effectively delivered to endemic populations. Prospects for LF elimination should be even brighter if MDA can be integrated with distribution of insecticide-treated bednets [11].
